# Flavanol Glycoside Content of Grape Seeds and Skins of *Vitis vinifera* Varieties Grown in Castilla-La Mancha, Spain

**DOI:** 10.3390/molecules24214001

**Published:** 2019-11-05

**Authors:** José Pérez-Navarro, Guillaume Cazals, Christine Enjalbal, Pedro Miguel Izquierdo-Cañas, Sergio Gómez-Alonso, Cédric Saucier

**Affiliations:** 1 IRICA, Univ. Castilla-La Mancha, 13071 Ciudad Real, Spain; jose.pnavarro@uclm.es (J.P.-N.); sergio.gomez@uclm.es (S.G.-A.); 2 IBMM, Univ Montpellier, CNRS, ENSCM, 34000 Montpellier, France; guillaume.cazals@umontpellier.fr (G.C.); christine.enjalbal@umontpellier.fr (C.E.); 3 IVICAM-IRIAF, 13700 Tomelloso, Spain; pmizquierdo@jccm.es; 4 PCTCLM, 02006 Albacete, Spain; 5 SPO, Univ Montpellier, INRA, Montpellier Supagro, 34000 Montpellier, France

**Keywords:** phenolic compounds, flavan-3-ol, glycoside, grape, *Vitis vinifera*, liquid chromatography, mass spectrometry

## Abstract

Glycosylated flavanols (monoglycosides and diglycosides) in skin and seed extracts of *Vitis vinifera* grapes grown in Castilla-La Mancha (Spain) were investigated using ultra-high-performance liquid chromatography—tandem mass spectrometry (UHPLC-ESI-QQQ-MS/MS). Six grape varieties (Airén, Tempranillo, the recently identified Albillo Dorado, Montonera del Casar, Moribel, and Tinto Fragoso) were studied over two consecutive years (2016 and 2017). A total of twenty monomeric flavanol monoglycosides, four diglycosylated monomers, and three dimeric flavanol monoglycosides were detected in all grape samples. The diversity observed in the composition of glycosylated flavanol in the grape berries suggests a strong influence of variety and grape tissue (skin or seed). Monomeric flavanol glycosides were more abundant in grape seed extracts, in contrast with monoglycosylated dimeric forms. In addition, the glycosylated flavanol content was related to berry color in grape skins, with higher concentrations measured in black grape varieties.

## 1. Introduction

Flavanols are bioactive secondary metabolites of plants and quantitatively are the most abundant flavonoid class of phenolic compounds found in grape berries [1,2]. The chemical structure of these compounds is characterized by a three-ring C6-C3-C6 carbon skeleton, (“flavan”) comprising a saturated central heterocycle ring with an oxygen and a hydroxyl group at the C3 position (“-ol”). The principal flavanol monomers found in grapes are (+)-catechin and its enantiomer (−)-epicatechin, followed by (−)-epigallocatechin and the galloylated form of (−)-epicatechin (i.e., (−)-epicatechin gallate), which is found at lower concentrations. These compounds are mostly located in grape skins and seeds [3,4,5].

Grape variety is strongly related to the biosynthesis of flavanols, showing a particular pattern of flavanols which can be a useful tool for differentiating varieties [6]. In addition to genetic influence, the concentration of flavanols in grapes is dependent on other external factors, such as environmental conditions (including temperature, light, nutrient and water status, soil type, vegetative growth, yield, and viticultural practices [7,8]).

These compounds exist not only as monomers, but also as tannin polymers, of which there are two classes: hydrolysable tannins (gallotannins and ellagitannins, that are polymers formed by gallic and ellagic acids, respectively) and proanthocyanidins or condensed tannins, that are polymers of flavanols [9], the latter group occurring naturally in grape berries.

Flavanols play a role in the quality of red wines, since they are responsible of wine sensory attributes such as astringency, due to their ability to precipitate salivary proteins in the oral cavity. Flavanols are also involved in color stability through copigmentation reactions [10,11] and the formation of new pigment compounds with different spectroscopic attributes [12,13,14,15,16].

Glycosylated hexoside forms of flavanols have been described in Merlot, Syrah, Tannat, and Cabernet Sauvignon grapes [17,18], of which a substantial number consisted of monoglycoside and diglycoside derivatives of flavanol monomers and dimers, highlighting the diversity of these compounds. The concentration of glycosylated flavanols in grapes depends on variety and maturity, and where they occur in the berries [18,19]. During ripening, the concentration of monomeric flavanol monoglycosides decreases and those of diglycoside forms increase until veraison is reached [19]. The role of flavanol glycosides in plant metabolism remains to be elucidated, but studies suggest they might contribute to the formation of proanthocyanidins [20,21] and behave as markers of seed or skin extraction during fermentation [18].

**Figure 1:**
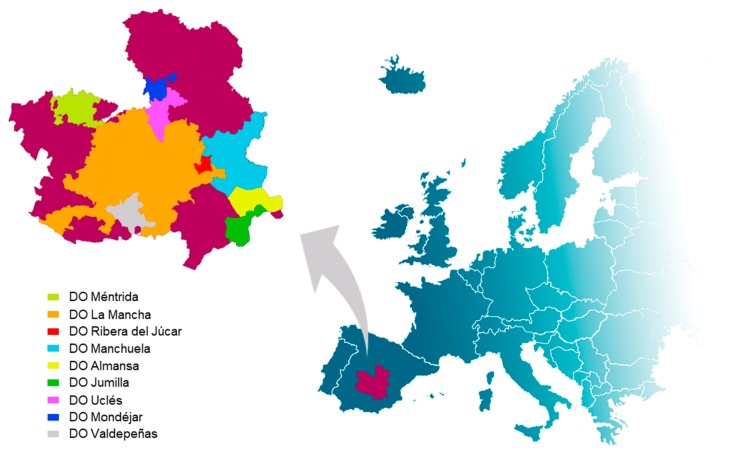
Wine designations of origin (DOs) in the Spanish region of Castilla-La Mancha.

The Castilla-La Mancha grape growing region in Spain is considered to be the largest wine growing area worldwide. It covers more than 470,000 hectares with nine wine designations of origin (DOs) (Figure 1). The Castilla-La Mancha wine heritage includes several varieties and novel grape genotypes (e.g., Albillo Dorado, Montonera del Casar, Moribel, and Tinto Fragoso) which were recently genetically identified and not previously registered in any database [22].

Given the importance of phenolic compounds and the limited data published for novel grape genotypes grown in Castilla-La Mancha, the goal of this study was to determine the content of flavanol glycosides of *Vitis vinifera* grape varieties grown in this Spanish region. These compounds were studied in both grape skins and seeds for 2016 and 2017 vintages using ultra-high-performance liquid chromatography coupled to tandem mass spectrometry (UHPLC-ESI-QQQ-MS/MS) in multiple reaction monitoring (MRM).

## 2. Results and Discussion

Several glycosylated flavanol monomers and dimers were identified and quantified in skins and seeds of the *Vitis vinifera* grapes. These compounds can be divided into three distinct groups: monomeric flavanol monoglycosides (MMG), monomeric flavanol diglycosides (MDG), and dimeric flavanol monoglycosides (DMG). Results for each group are discussed in the following sections.

### 2.1. Monomeric Flavanol Monoglycosides (MMG) in Grape Skins and Seeds

#### 2.1.1. MMG Identification

Monomeric flavanol monoglycosides were identified by the ion fragments produced in MS/MS. The signals attributable to catechin or epicatechin monoglycosides were detected at *m*/*z* 451 and the MS/MS fragmentation patterns showed that the loss of the B ring resulted in the most abundant fragment ion (*m*/*z* 341). Therefore, the [451 → 341] transition was selected to identify and quantify these compounds (Table S1). An ion fragment at *m*/*z* 289 was also observed corresponding to an (epi)catechin unit after the loss of a hexose attached to the hydroxyl group.

**Figure 2:**
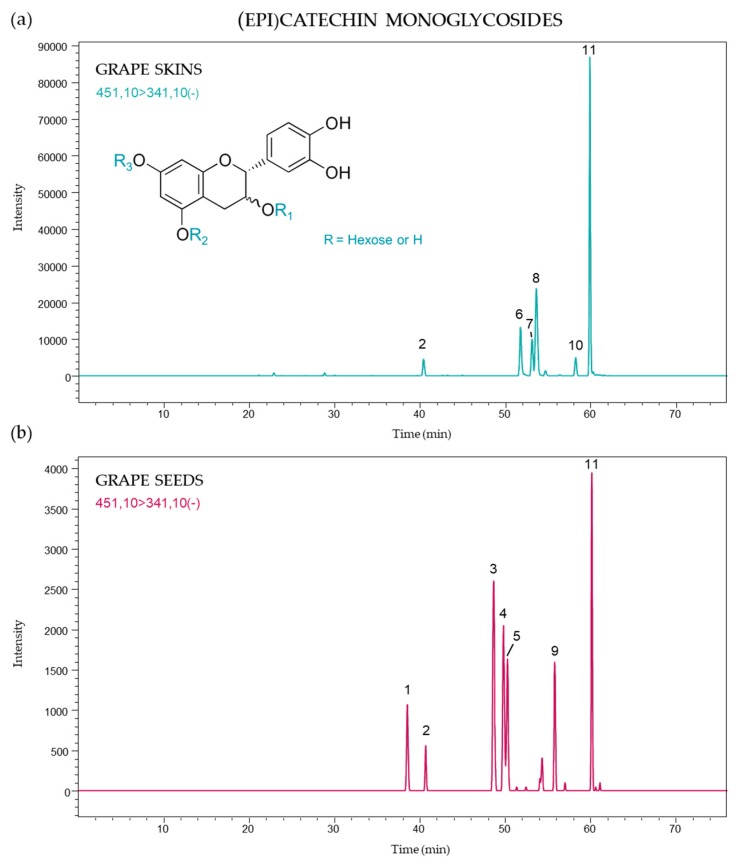
(Epi)catechin monoglycoside profile of (**a**) skins and (**b**) seeds from *Vitis vinifera* grapes using multiple reaction monitoring (MRM) [451 → 341].

A total of eleven (epi)catechin monoglycosides were identified in grape skins and seeds, probably corresponding to isomers with a hexose (glucose or galactose) moiety linked to different hydroxyl groups on the flavanol core, and the presence of these compounds differed according to grape part and variety. Figure 2 shows the (epi)catechin monoglycoside profile in both skins and seeds. Six and seven monoglycosylated derivatives of (epi)catechin were identified in skins and seeds, respectively, and only compounds 2 and 11 were found in both grape parts. The strongest signal was attributed to peak 11 (60.1 min) for all samples.

These types of compounds were previously reported for Merlot, Tannat, and Syrah grapes, where ten (epi)catechin monodiglycosides were identified in skins and seeds [19].

**Figure 3:**
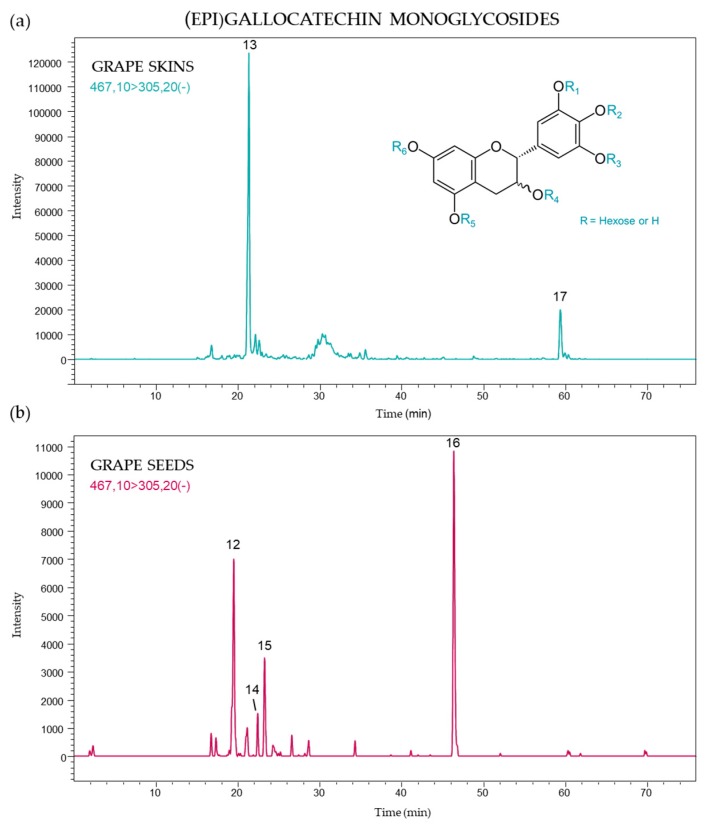
(Epi)gallocatechin monoglycoside profile of (**a**) skins and (**b**) seeds from *Vitis vinifera* grapes using MRM [467 → 305].

Other monoglycosylated flavanol monomers were identified as (epi)gallocatechin derivatives. A deprotonated molecular ion ([M − H]^−^) at *m*/*z* 467 was found for these compounds with a typical fragmentation signal at *m*/*z* 305 corresponding to the ion of a deprotonated monomeric (epi)gallocatechin unit resulting from the loss of a hexose moiety. In this study, six compounds were detected, and Figure 3 presents the (epi)gallocatechin monoglycosides found in both grape skins and seeds using the targeted MS/MS [467 → 305] transition. The (epi)gallocatechin MS/MS profile showed different isomers according to their localization in the berry (Figure 3). Peak 13 (21.2 min) and 17 (59.4 min) were only identified in skins, while the compound corresponding to peak 13 occurred at higher concentrations in all samples. The profiles of (epi)gallocatechin monoglycoside in seeds were characterized by greater signals of peak 16 (45.9 min) and peak 12 (19.4 min).

**Figure 4:**
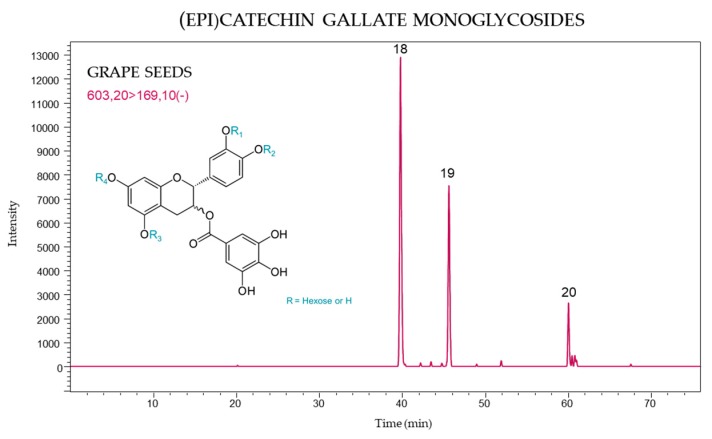
(Epi)catechin gallate monoglycoside profile of *V. vinifera* grape seeds using MRM [603 → 169].

The molecular ion at *m*/*z* 603 was attributed to the monomeric glycoside flavanol (epi)catechin gallate, and the [603 → 169] transition in MS/MS, used for greater selectivity, yielded a fragment at *m*/*z* 169 corresponding to the loss of gallic acid. It was possible to identify three monomers of (epi)catechin gallate monoglycosides which were detected only in seeds (Figure 4). Peaks 18 (39.8 min) and 19 (45.7 min) were present in all grape varieties. These compounds, together with the (epi)gallocatechin monoglycosides were previously identified and described in seed extracts of Merlot grapes [23].

#### 2.1.2. MMG Quantification

**Figure 5:**
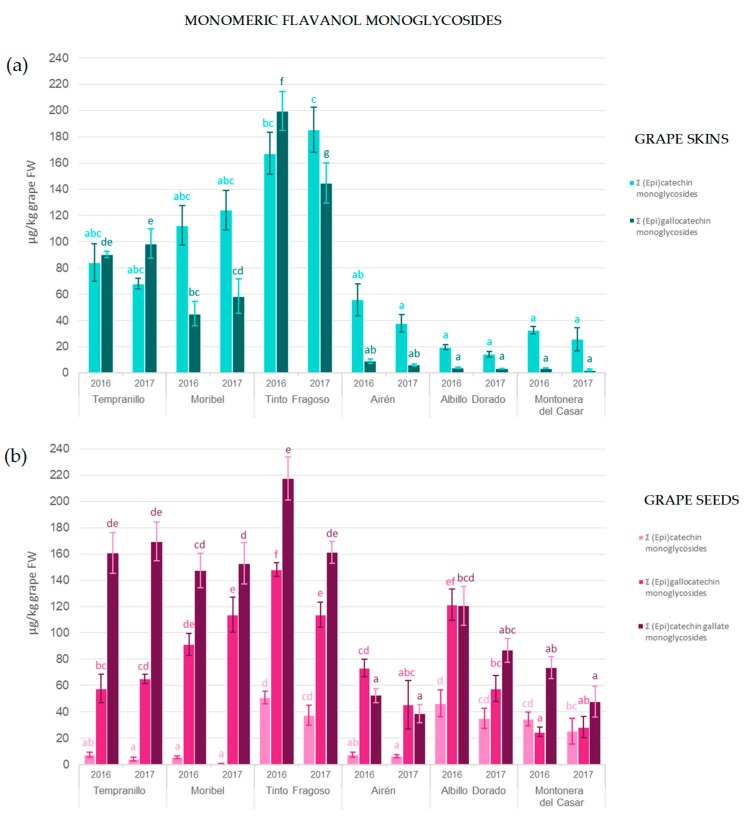
Contents of different monomeric flavanol monoglycosides found in (**a**) skin and (**b**) seed extracts from *Vitis vinifera* grape varieties.

The total concentrations of different monomeric flavanol monoglycosides in skin and seed extracts are shown in Figure 5.

The concentration of (epi)catechin monoglycosides ranged from 1 to 51 µg/kg grape fresh weight (FW) in seeds and 14–185 µg/kg grapes FW for skins. Furthermore, substantial differences in the concentration of these compounds were found among varieties investigated. Black grape skins had higher concentrations than the skins of the white varieties; the Tinto Fragoso genotype was richest in both 2016 and 2017 containing, respectively, 167 and 185 µg/kg grape FW. The seeds of Albillo Dorado and Tinto Fragoso grapes showed higher concentrations of (epi)catechin monoglycoside. Generally, the binding of a sugar moiety to the monomeric (epi)catechin unit stabilizes its chemical structure and renders it more stable at pH values from 4 to 8 [24].

Greater concentrations of (epi)gallocatechin monoglycosides were found in seeds compared to skins and this was particularly the case for white grapes. Tinto Fragoso grapes contained the highest concentrations of these compounds in 2016 (200 and 148 µg/kg grape FW in grape skins and seeds, respectively). (Epi)catechin gallate monoglycosides could be quantified only in grape seeds, this being one of the main differences in the MMG profile of both grape tissues. The concentration of (epi)catechin gallate monoglycoside ranged from 39 to 218 µg/kg grape FW, with lower levels found in the seeds of white as compared to black grape varieties.

The total concentration of MMG’s in skins and seeds are shown in Table 1, which shows that concentrations differed between skins and seeds; ranging from 90 to 417 µg/kg grape FW for seeds, with values greater than the skin total content of 18–367 µg/kg grape FW. Tinto Fragoso contained the highest concentration of MMG’s in both skins and seeds over the two consecutive vintages. Statistically significant differences were found between the vintages for some samples, with concentrations generally higher in 2016. This may be due to the effect of external factors such as environmental conditions which affect the biosynthesis of flavonoids [7]. Furthermore, previous studies reported that the MMG concentration depends on the degree of berry maturity, where the concentration of monomeric flavanol monoglycosides decreases during grape ripening [19].

### 2.2. Monomeric Flavanol Diglycosides (MDG) in Grape Skins and Seeds

#### 2.2.1. MDG Identification

**Figure 6:**
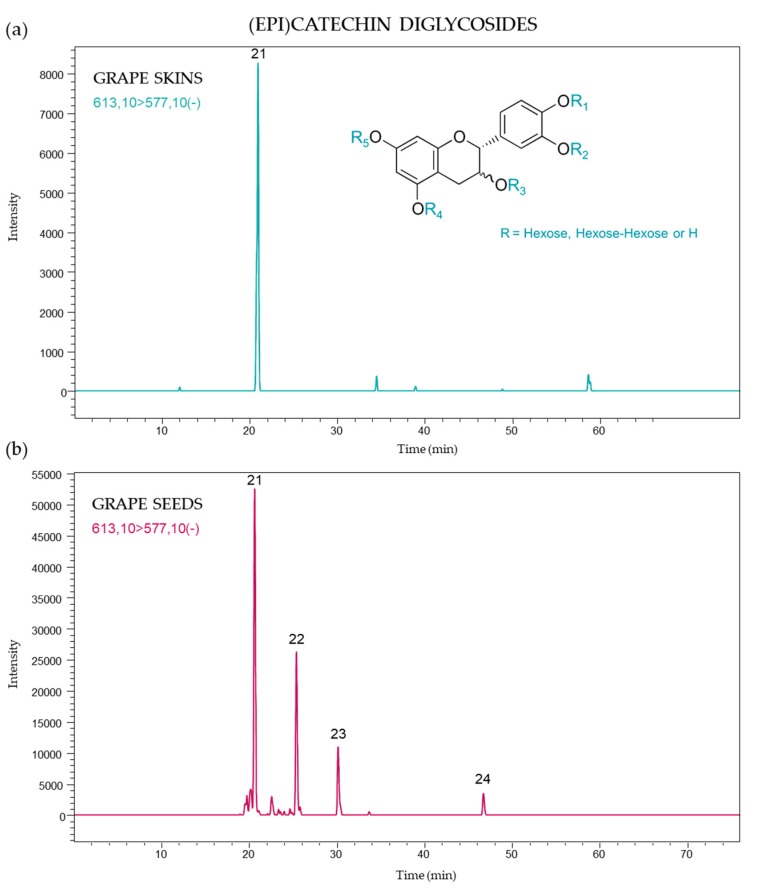
(Epi)catechin diglycoside profile of (**a**) skins and (**b**) seeds from *Vitis vinifera* grapes using MRM [613 → 577].

Flavanol monomers linked to two hexose moieties (diglycosides) were also found in the grape varieties studied. The signals attributable to catechin or epicatechin diglycosides were detected at *m*/*z* 613, and the principal MS/MS ion products were at *m*/*z* 577 (loss of two water molecules) and at *m*/*z* 289 (which corresponds to a neutral loss of two hexose units), which indicates that the monomeric unit is (epi)catechin in this instance. These compounds were quantified using the targeted transition [613 → 577], and four compounds were found corresponding to isomers of (epi)catechin monomers bearing two hexose units linked to hydroxyl groups at different positions or two hexoses linked to the flavanol core at the same position (hexosyl-hexoside). An example of an MDG ion chromatogram of grape skins and seeds is shown in Figure 6.

A total of nine MDG’s were previously reported in seeds of several grape varieties, [19]. However, in this study one of the four compounds previously identified was also detected in grape skins, i.e., peak 21 (20.7 min.). This type of compound was previously described for Grenache and Syrah grapes, but in seeds only [18]. The MDG’s compounds corresponding to peaks 21–24 were found in the seeds of all samples. This is not the first time that phenolic diglycosides were identified in grapes: diglycosylated anthocyanins were used as varietal markers of non-*V. vinifera* and hybrid grapes [25,26], dominating the anthocyanin profile [27,28,29]. Nevertheless, trace concentrations of anthocyanin diglucosides were also found in *V. vinifera* grapes [30,31,32,33].

#### 2.2.2. MDG Quantification

The first eluted MDG (peak 21) was the most abundant in all samples. Substantial differences were found between both berry parts, with concentrations in the range 36–257 µg/kg grape FW for seeds, and less than 10 µg/kg grape FW in almost all skin samples (Table 2). Peak 22 (25.4 min) was the second most abundant MDG found in seeds (13–207 µg/kg grape FW).

The total concentrations of MDG’s in grape seeds ranged from 56 to 494 µg/kg grape FW; which were greater than those found in skins (1–15 µg/kg grape FW). These results concur with those previously reported for Grenache and Syrah varieties (0.5–1 mg/kg seeds and 38–48 µg/kg skins) [18]. In general, total MDG’s were more abundant in seeds than the skins of grapes harvested in 2016. The concentration of MDG’s is also influenced by grape maturity, though contrary to MMG’s, concentrations increase in skins during berry ripening [19].

### 2.3. Dimeric Flavanol Monoglycosides (DMG) in Grape Skin and Seed Extracts

#### 2.3.1. DMG Identification

**Figure 7:**
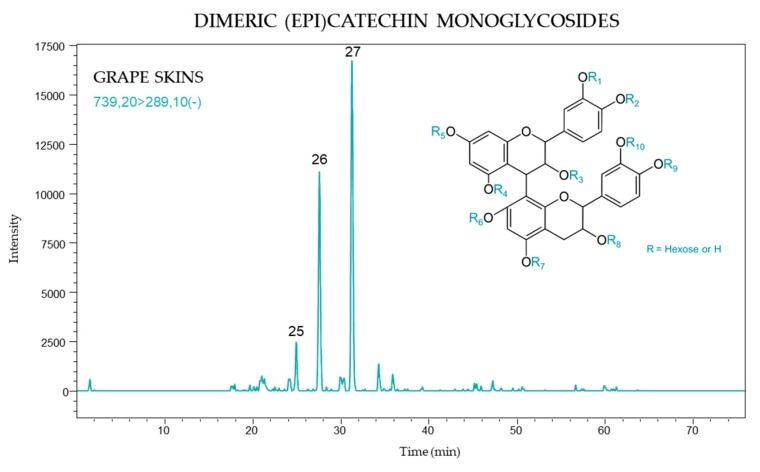
Dimeric (epi)catechin monoglycoside (DMG) profile of skins from *Vitis vinifera* grapes using MRM [739 → 289].

The targeted [739 → 289] transition was used to detect DMG’s as they present a molecular ion at *m*/*z* 739 and a fragment ion at *m*/*z* 289, corresponding to a neutral loss of an (epi)catechin-monoglucoside unit. The MRM ion chromatogram obtained for these compounds in grape skin is presented in Figure 7.

Three monoglycosylated derivatives of flavanol dimers were identified in grape skins only. The retention times of these compounds were 24.8, 27.6, and 31.2 min., the latter peak giving the most intense signal in all samples (peak 27).

#### 2.3.2. DMG Quantification

Statistically significant differences in the concentration of each identified DMG were observed between black and white grapes. The white varieties, Airén, Albillo Dorado, and Montonera del Casar, had lower levels of each DMG compound as compared to black grapes, and moreover peak 25 was not detected in white varieties (Table 3). The most abundant DMG corresponded to peak 27, with values ranging from 3 to 47 µg/kg grape FW, followed by peak 26 (2–25 µg/kg grape FW).

DMG’s were found in skins only, although it was previously shown that these compounds occurred in the seeds of some grape varieties [18]. However, their content decreased during ripening with only trace concentrations at maturity [19]. The total content of DMG’s ranged from 5 to 77 µg/kg grape FW in these samples, with Tinto Fragoso accounting for the highest concentration of DMG’s in both 2016 (69 µg/kg grape FW) and 2017 (77 µg/kg grape FW). However, the differences between vintages were not statistically significant with respect to the concentration of DMG’s.

## 3. Materials and Methods

### 3.1. Chemicals

All chemicals used for the extraction of the phenolic compounds, i.e., methanol and formic acid, were of analytical grade (>99%, Fisher Scientific, Madrid, Spain) and water was obtained from the Milli-Q system (Merck-Millipore, Darmstadt, Germany). For the UHPLC analysis of flavanol glycosides, formic acid and methanol solvents were supplied by Sigma Aldrich (St. Louis, MO, USA) and Milli-Q water was obtained from Milli-Q system (Millipore, Molsheim, France). (+)-Catechin-4′-*O*-*β*-glucoside was hemisynthesized as previously described [17] from (+)-catechin commercial standard supplied by Sigma Chemical Company (St. Louis, MO, USA).

### 3.2. Grape Samples

Grape samples of six *Vitis vinifera* varieties, three white (Airén, Albillo Dorado, and Montonera del Casar) and three black (Moribel, Tempranillo, and Tinto Fragoso), were harvested at optimum ripeness for winemaking (19–24º Brix) in 2016 and 2017 vintages. All grapes were grown in an experimental vineyard located in the Instituto Regional de Investigación y Desarrollo Agroalimentario y Forestal (IRIAF) of Castilla-La Mancha region (central southern Spain) under the same conditions (soil type, climate and cultivation practices). For each grape variety, samples were randomly collected in three batches from 10 different vines located in three different vineyard zones. Laboratory samples were obtained by random selection of berries from different parts of the clusters (top, central, and bottom) and 100 berries were selected from each of the three batches, obtaining three replicates.

### 3.3. Extraction of Phenolic Compounds

Grapes were carefully peeled by hand and skins and seeds were separated manually, washed in water and gently dried with filter paper. Subsequently, they were freeze-dried for 24 h (Cryodos-50/230 V 50 Hz Telstar, Madrid, Spain) and mill-crushed (IKA A10 basic, A 10.2 Star shaped cutter, Staufen, Germany) at 25,000 rpm for 10 s (skins) and 2 min (seeds) in order to obtain a fine powder. Extracts of phenolic compounds from grape skins and seeds were obtained as previously described [32], using an accurately-weighed amount of powder (~1 g skins and ~0.4 g seeds) and 25 mL methanol/water/formic acid (50:48.5:1.5 *v*/*v*/*v*). Extraction was carried out at 4 °C using a Qsonica Q500 sonicator (Cole-Parmer, Vernon Hills, IL, USA) using 80% of output amplitude, with the duty cycle of 15 s turned on and 5 s off. The extracts were then centrifuged at 5000 rpm for 5 min at 5 °C (Beckman Coulter Avanti J-26 XP; JA-14 rotor, Life Sciences, Indianapolis, IN, USA). Two extractions were necessary to obtain a yield of approximately 99% of the extractable phenolic content [29]. Phenolic fractions were stored at −20 °C until analysis.

### 3.4. UHPLC-ESI-QQQ-MS/MS Analysis of Flavanol Glycosides

The identification and quantitation of glycosylated flavanols was carried out on a Nexera X2 UHPLC system (Shimadzu, Marne la Vallée, France) coupled to a Shimadzu LCMS-8050 triple quadrupole mass spectrometer in multiple reaction monitoring (MRM), following a previously described method [19]. Extracts of grape skins and seeds were centrifuged before injecting a volume (10 µL skins, 5 µL seeds) on two Zorbax SB-C18 reversed-phase columns in series (2.1 × 150 mm and 2.1 × 100 mm; 1.8 µm dp) supplied by Agilent Technologies (Santa Clara, CA, USA). Separations were carried out at 40 °C, with a flow rate of 0.35 mL/min. Solvent A (water, 0.1% formic acid) and solvent B (methanol, 0.1% formic acid) were used with a linear gradient as follows: 0 min (0% B), 7–17 min (0–18% B), 17–57 min (18–33% B), 57–58 min (40–100% B), 58–67 min (100% B), 67–68 min (100–0% B), 68 min (0% B). Mass spectrometry conditions were as follows: negative ionization mode; electrospray interface voltage, 3 kV; heat block temperature, 350 °C; interface temperature, 300 °C; desolvation line temperature, 300 °C; dry gas, 5 L/min; nebulizing gas, 3 L/min; and heating gas, 15 L/min. Flavanol glycosides were quantified by interpolation on a linear regression calibration curve of (+)-catechin-4′-*O*-*β*-glucoside standard (in the concentration range 0.01–5 mg/L, Table S2). The concentration of flavanol glycosides were expressed as (+)-catechin-4′-*O*-*β*-glucoside equivalents.

### 3.5. Statistical Analysis

Data were analyzed by a one-way analysis of variance (ANOVA, Tukey’s test, *p* < 0.05) using SPSS software version 23.0 (SPSS Inc., Chicago, IL, USA).

## 4. Conclusions

This work contributes to the characterization of flavanol glycosides present in skins and seeds of *Vitis vinifera* grape varieties grown in Castilla-La Mancha (Airén, Tempranillo, and four recently identified grape genotypes). The composition of glycosylated flavanols UHPLC-ESI-MS/MS in MRM mode allowed the quantification of dimeric flavanol monoglycosides in addition to several monomeric flavanol mono- and diglycosides.

Flavanol glycoside profiles of these grapes were characterized by a higher total content of glycosylated monomers in seeds, a significant proportion of which comprised monoglycosylated derivatives of (epi)catechin gallate. Similar profiles were observed for monomeric flavanol diglycosides, which were also more abundant in seeds, whereas monoglycosylated dimers were present exclusively in skins. Moreover, the concentration of glycosylated flavanols was greater in black varieties. The profile and contents of flavanol glycosides varied according to the berry part and variety, but also significant differences were found among vintages, mainly in the MDG concentrations. These preliminary results broaden current knowledge relating of the characterization and location of flavanol glycosides in grape berries. Additional studies using other grape species (non-*V. vinifera* or hybrids) grown under different conditions would further the understanding of these compounds and their role in different grape varieties. Furthermore, differences in the distribution of flavanol glycosides among the varieties and between vintages indicate that these compounds might serve as useful markers for seed or skin extraction during winemaking, given their contribution to the formation of proanthocyanidins and their impact on wine sensory properties, such as astringency.

## Figures and Tables

**Table 1:** Total monomeric flavanol monoglycosides (MMG) of skin and seed extracts from *Vitis vinifera* grape varieties (mean value ± standard deviation, *n* = 3), µg/kg of grape FW.

Tempranillo		Moribel		Tinto Fragoso		Airén		Albillo Dorado		Montonera del Casar	
2016	2017	2016	2017	2016	2017	2016	2017	2016	2017	2016	2017
**Seeds**											
225.94 ± 59.84 ^bc^	238.80 ± 32.72 ^bc^	244.27 ± 18.74 ^bc^	267.58 ± 28.81 ^bc^	416.59 ± 30.40 ^d^	312.47 ± 9.82 ^c^	132.93 ± 9.85 ^a^	90.46 ± 25.98 ^a^	288.58 ± 33.20 ^c^	179.37 ± 26.87 ^ab^	132.97 ± 8.87 ^a^	101.83 ± 36.18 ^a^
**Skins**											
174.64 ± 19.29 ^cd^	166.71 ± 15.09 ^bcd^	157.66 ± 31.75 ^abcd^	182.71 ± 49.14 ^de^	366.88 ± 60.11 ^f^	330.04 ± 98.39 ^ef^	64.88 ± 13.74 ^abcd^	44.18 ± 7.31 ^abcd^	23.65 ± 2.06 ^ab^	17.65 ± 2.60 ^a^	36.30 ± 2.79 ^abcd^	28.14 ± 9.49 ^abc^

Different letters in the same row indicate significant differences (ANOVA, Tukey’s test, *p* < 0.05).

**Table 2:** Individual and total monomeric flavanol diglycosides (MDG) of skin and seed extracts from *Vitis vinifera* grape varieties, expressed as µg/kg grape FW (mean value ± standard deviation, *n* = 3).

	Tempranillo		Moribel		Tinto Fragoso		Airén		Albillo Dorado		Montonera del Casar	
MDG	2016	2017	2016	2017	2016	2017	2016	2017	2016	2017	2016	2017
**Peak**	**Seeds**											
21	196.32 ± 31.45 ^def^	36.01 ± 11.21 ^a^	243.07 ± 26.19 ^ef^	48.95 ± 2.99 ^ab^	257.23 ± 26.19 ^f^	42.84 ± 3.56 ^a^	127.56 ± 20.58 ^bcd^	48.87 ± 12.15 ^ab^	147.02 ± 25.81 ^cd^	107.71 ± 34.10 ^abc^	91.80 ± 8.22 ^abc^	166.48 ± 56.83 ^cde^
22	142.75 ± 43.65 ^cd^	16.36 ± 2.88 ^a^	206.96 ± 37.56 ^d^	16.51 ± 3.33 ^a^	126.79 ± 19.78 ^bc^	13.01 ± 4.17 ^a^	68.83 ± 6.40 ^ab^	28.26 ± 18.57 ^a^	138.97 ± 26.93 ^cd^	123.97 ± 31.37 ^bc^	48.68 ± 8.68 ^a^	60.66 ± 15.01 ^ab^
23	32.45 ± 9.91 ^d^	2.81 ± 1.11 ^a^	27.57 ± 1.31 ^cd^	3.24 ± 0.76 ^a^	56.68 ± 13.34 ^e^	6.53 ± 1.71 ^ab^	22.11 ± 6.72 ^bcd^	3.92 ± 2.20 ^a^	16.97 ± 7.57 ^abcd^	10.09 ± 2.42 ^ab^	11.56 ± 1.35 ^abc^	21.37 ± 4.11 ^bcd^
24	19.10 ± 4.98 ^b^	1.29 ± 0.17 ^a^	16.01 ± 3.59 ^b^	2.93 ± 0.71 ^a^	18.09 ± 3.32 ^b^	3.24 ± 1.03 ^a^	3.64 ± 0.95 ^a^	1.81 ± 0.56 ^a^	3.04 ± 1.21 ^a^	12.65 ± 2.55 ^b^	1.00 ± 0.49 ^a^	4.05 ± 1.90 ^a^
**Total**	390.63 ± 87.37 ^def^	56.47 ± 13.37^a^	493.61 ± 61.72 ^f^	71.64 ± 1.55 ^ab^	458.80 ± 70.41 ^ef^	65.62 ± 9.79 ^ab^	222.15 ± 33.15 ^bc^	82.85 ± 30.92 ^ab^	306.01 ± 57.40 ^cde^	254.34 ± 54.25 ^cd^	153.05 ± 16.67 ^abc^	252.57 ± 67.43 ^cd^
**Peak**	**Skins**											
21	7.16 ± 1.65 ^bcd^	9.38 ± 1.76 ^d^	7.30 ± 1.55 ^cd^	9.29 ± 1.41 ^d^	7.21 ± 1.77 ^cd^	15.07 ± 5.12 ^e^	1.70 ± 0.28 ^ab^	2.99 ± 0.76 ^abc^	2.81 ± 1.02 ^abc^	2.08 ± 0.48 ^abc^	1.36 ± 0.52 ^a^	1.97 ± 0.11 ^abc^
**Total**	7.16 ± 1.65 ^bcd^	9.38 ± 1.76 ^d^	7.30 ± 1.55 ^cd^	9.29 ± 1.41 ^d^	7.21 ± 1.77 ^cd^	15.07 ± 5.12 ^e^	1.70 ± 0.28 ^ab^	2.99 ± 0.76 ^abc^	2.81 ± 1.02 ^abc^	2.08 ± 0.48 ^abc^	1.36 ± 0.52 ^a^	1.97 ± 0.11 ^abc^

Different letters in the same row indicate significant differences (ANOVA, Tukey’s test, *p* < 0.05).

**Table 3:** Individual and total dimeric flavanol monoglycosides (DMG) of skin extracts from *Vitis vinifera* grape varieties, expressed as µg/kg grape FW (mean value ± standard deviation, *n* = 3).

	Tempranillo		Moribel		Tinto Fragoso		Airén		Albillo Dorado		Montonera del Casar	
DMG Peak	2016	2017	2016	2017	2016	2017	2016	2017	2016	2017	2016	2017
25	3.05 ± 0.81 ^a^	3.06 ± 1.24 ^a^	3.56 ± 0.93 ^a^	3.89 ± 0.50 ^a^	5.35 ± 1.12 ^ab^	6.96 ± 2.16 ^b^	ND	ND	ND	ND	ND	ND
26	12.92 ± 1.47 ^e^	8.44 ± 1.14 ^abc^	18.62 ± 5.37 ^cde^	17.39 ± 3.49 ^cde^	25.41 ± 6.01 ^e^	22.18 ± 8.12 ^de^	6.09 ± 1.97 ^ab^	5.45 ± 0.24 ^ab^	2.45 ± 0.66 ^ab^	2.01 ± 2.60 ^a^	3.75 ± 0.62 ^ab^	2.86 ± 0.73 ^ab^
27	18.41 ± 1.83 ^abc^	22.21 ± 3.66 ^bcd^	29.89 ± 9.90 ^cd^	29.85 ± 7.08 ^cd^	37.86 ± 4.86 ^de^	47.43 ± 13.49 ^e^	8.15 ± 1.61 ^ab^	7.15 ± 1.37 ^ab^	4.45 ± 1.54 ^a^	2.71 ± 1.10 ^a^	7.51 ± 1.30 ^ab^	5.51 ± 2.39 ^ab^
**Total**	34.38 ± 4.11 ^bc^	33.72 ± 5.92 ^bc^	52.09 ± 15.92 ^cd^	51.14 ± 10.84 ^cd^	68.62 ± 11.41 ^d^	76.57 ± 23.69 ^d^	14.24 ± 3.55 ^ab^	12.59 ± 1.48 ^ab^	6.89 ± 2.19 ^ab^	4.71 ± 1.61 ^a^	11.26 ± 1.82 ^ab^	8.38 ± 3.06 ^ab^

Different letters in the same row indicate significant differences (ANOVA, Tukey’s test, *p* < 0.05). Abbreviations: ND, not detected.

## Supplementary Materials

The following are available online. Table S1: MS/MS chromatographic retention time (RT) and transitions of flavanol glycosides identified in *V. vinifera* grape skins and seeds, Table S2: Calibration data of (+)-catechin-4′-*O*-*β*-glucoside standard used for the MS/MS quantification of flavanol glycosides in *Vitis vinifera* grape skins and seeds.
