# Supplementary material for: Flavanol Glycoside Content of Grape Seeds and Skins of Vitis vinifera Varieties Grown in Castilla-La Mancha, Spain
Source: Molecules. 2019 Nov 5;24(21):4001. doi: 10.3390/molecules24214001 (PMC6864760; doi:10.3390/molecules24214001)
Supplement: Supplementary file 1 [file molecules-24-04001-s001.pdf]

Table S1. MS/MS chromatographic retention time (RT) and transitions of flavanol glycosides identified in *V. vinifera* grape skins and seeds.

Peak	Compound	RT (min)	Transition m/z
1	(Epi)catechin monoglycoside	38.4	451.10 > 341.10
2	(Epi)catechin monoglycoside	40.5	451.10 > 341.10
3	(Epi)catechin monoglycoside	48.5	451.10 > 341.10
4	(Epi)catechin monoglycoside	49.8	451.10 > 341.10
5	(Epi)catechin monoglycoside	50.4	451.10 > 341.10
6	(Epi)catechin monoglycoside	51.7	451.10 > 341.10
7	(Epi)catechin monoglycoside	53.1	451.10 > 341.10
8	(Epi)catechin monoglycoside	53.7	451.10 > 341.10
9	(Epi)catechin monoglycoside	55.8	451.10 > 341.10
10	(Epi)catechin monoglycoside	58.4	451.10 > 341.10
11	(Epi)catechin monoglycoside	60.1	451.10 > 341.10
12	(Epi)gallocatechin monoglycoside	19.4	467.10 > 305.20
13	(Epi)gallocatechin monoglycoside	21.2	467.10 > 305.20
14	(Epi)gallocatechin monoglycoside	22.4	467.10 > 305.20
15	(Epi)gallocatechin monoglycoside	23.2	467.10 > 305.20
16	(Epi)gallocatechin monoglycoside	45.9	467.10 > 305.20
17	(Epi)gallocatechin monoglycoside	59.4	467.10 > 305.20
18	(Epi)catechin gallate monoglycoside	39.8	603.20 > 169.10
19	(Epi)catechin gallate monoglycoside	45.7	603.20 > 169.10
20	(Epi)catechin gallate monoglycoside	60.0	603.20 > 169.10
21	(Epi)catechin diglycoside	20.7	613.10 > 577.10
22	(Epi)catechin diglycoside	25.4	613.10 > 577.10
23	(Epi)catechin diglycoside	30.1	613.10 > 577.10
24	(Epi)catechin diglycoside	46.7	613.10 > 577.10
25	(Epi)catechin dimer monoglycoside	24.8	739.20 > 289.10
26	(Epi)catechin dimer monoglycoside	27.6	739.20 > 289.10
27	(Epi)catechin dimer monoglycoside	31.2	739.20 > 289.10

Table S2. Calibration data of (+)-catechin-4′-O-β-glucoside standard used for the MS/MS quantification of flavanol glycosides in *Vitis vinifera* grape skins and seeds.

Compound	RT (min)	Transition m/z	Linear range (mg/L)	Linear curve	R^2	LOD (mg/L)	LOQ (mg/L)
(+)-Catechin-4′-O-β-glucoside	20.8	451.10 > 289.10	0.01 - 5	y = 23608951.71x + 46060.94	0.9997	0.022	0.074
